# Evaluating the effectiveness and safety of a novel phage cocktail as a biocontrol of *Salmonella* in biofilm, food products, and broiler chicken

**DOI:** 10.3389/fmicb.2024.1505805

**Published:** 2024-11-28

**Authors:** Narges Torkashvand, Haniyeh Kamyab, Parisa Aarabi, Ahmad Reza Shahverdi, Mohammad Amir Karimi Torshizi, Mohammad Reza Khoshayand, Zargham Sepehrizadeh

**Affiliations:** ^1^Department of Pharmaceutical Biotechnology, Faculty of Pharmacy and Biotechnology Research Center, Tehran University of Medical Sciences, Tehran, Iran; ^2^Faculty of Pharmacy, Tehran University of Medical Sciences, Tehran, Iran; ^3^Department of Poultry Sciences, Faculty of Agriculture, Tarbiat Modares University, Tehran, Iran; ^4^Department of Food and Drug Control, Faculty of Pharmacy, Tehran University of Medical Sciences, Tehran, Iran

**Keywords:** biocontrol, phage cocktail, *Salmonella*, biofilm, food products, broiler chickens

## Abstract

*Salmonella* is a foodborne pathogen of animal and public health significance. Considering the disadvantages of antibiotics or chemical preservatives traditionally used to eliminate this pathogen, attention has shifted, in recent years, toward biocontrol agents such as bacteriophages, used either separately or in combination to prevent food contamination. However, extensive use of phage-based biocontrol agents in the food industry requires further studies to ensure their safety and efficacy. In the present study, we investigated the effectiveness and safety of phage cocktail, a phage cocktail comprising three pre-characterized *Salmonella* phages (vB_SenS_TUMS_E4, vB_SenS_TUMS_E15 and vB_SenS_TUMS_E19). First, we performed an MTT [3-(4,5-dimethylthiazol-2-yl)-2,5 diphenyl tetrazolium bromide] assay on a human foreskin fibroblast cell line, in which the resulting high cell viability revealed the safety of the phage cocktail. Next, we performed a time-kill assay in which a 4 Log decline in bacterial levels was detected. Additionally, we utilized a colorimetric method to evaluate the anti-biofilm activity of phage cocktail, in which it proved more efficacious compared to the MIC and MBEC levels of the antibiotic control. Then, we assessed the ability of phage cocktail to eradicate *Salmonella* in different food samples, where it considerably reduced the bacterial count regardless of the temperature (4°C and 25°C). Lastly, we used broiler chickens as an animal model to measure the growth-promoting activity of phage cocktail. *Salmonella*-infected chickens orally treated with modified phage cocktail demonstrated no mortality and a significant increase in weight gain compared to the untreated group (*p* ≤ 0.0002). The study presents a novel research evaluating the effectiveness and safety of a phage cocktail as a biocontrol agent against *Salmonella* in various contexts, including biofilms, food products, and broiler chickens. This multifaceted approach underscores the promising role of phage therapy as a sustainable biocontrol strategy in food safety and public health contexts.

## Introduction

*Salmonella* is a major foodborne pathogen that poses a global public health concern. Non-typhoidal *Salmonella*-induced gastroenteritis is so prevalent that as many as 93 million people are estimated to suffer from it annually around the world (Shaji et al., 2023a). Among the various *Salmonella* serovars, *Salmonella* Enteritidis is the most commonly reported *Salmonella* serovar in non-typhoidal salmonellosis, making up 65% of all cases (Khan and Rahman, 2022). This bacterium is found in a wide range of foods, including chicken, eggs, fruits, vegetables, and processed foods, with poultry products being the primary source of infection (World Health Organization, 2016). It is noteworthy that, in recent years, several outbreaks of salmonellosis have occurred rooting from fresh vegetables and fruit contamination (Whitworth, 2023; Teklemariam et al., 2023; Johnston, 2023). Hence, it is imperative to apply approaches to prevent *Salmonella* contamination of foods during production, processing, and storage. In recent years, the use of biocontrol agents to limit bacterial growth in the food chain has gained attention as alternatives to chemical preservatives and antibiotic-like compounds. Antibiotics are widely used in food production to improve animal growth. However, the extensive use of antibiotics of human significance in the food industry has created an ideal breeding ground for antibiotic resistance. Bacteriophages, viruses that merely infect bacteria, are perfectly suited to replace antibiotics as safe and potent antimicrobials (Xu, 2021). Broader and more effective activity against the targeted bacteria is achieved if, rather than a single type of phage, a combination of multiple phages (phage cocktail) is utilized. *Salmonella* phage cocktails have been shown to reduce the presence of this bacterium in fresh produce, meat, and dairy products (Lavilla et al., 2023). Moreover, Phage cocktails can disrupt *Salmonella* biofilms, which are bacterial communities attached to a surface and encapsulated in a protective matrix. Biofilms are of great concern in food production because they can survive harsh sanitation practices and provide a reservoir for microbial contamination. Studies have shown that *Salmonella* phages can effectively decrease biofilm viability (Gong and Jiang, 2017). This application of phages not only ensures food safety, but also provides a more sustainable method for controlling spoilage organisms compared to the use of chemical sanitizers. *Salmonella* not only poses a threat to human health but also affects the growth rate of birds, potentially reducing feed intake by up to 29% in broilers (Clavijo et al., 2022). Therefore, *Salmonella* phage cocktails have been studied in broiler chickens as animal models to assess the effectiveness of phage therapies in reducing *Salmonella* colonization and shedding. Studies have shown that phages can effectively reduce *Salmonella* levels in the chicken gut and cecum, indicating their potential as biocontrol agents for poultry production (Thanki et al., 2021).

As mentioned above, various studies have highlighted the effectiveness of *Salmonella* phage cocktails in food, biofilms, and animal models. Although these findings are promising, to successfully implement such phage cocktails as biocontrol agents and potentially integrate them into existing food safety protocols, it is crucial to evaluate their safety and efficacy in diverse settings. As a result, further research is necessary to optimize phage cocktails, assess their long-term impact on microbial communities, and ensure their safety for human consumption.

In the current study, we explored the efficacy and safety of phage cocktail, a phage cocktail consisting of three genetically and behaviorally characterized *S. enteritidis* lytic bacteriophages. The phages in the cocktail have undergone a thorough analysis, including host range determination, infection assays, stability under different environmental conditions, genome sequencing, electron microscopy, and safety assessments in previous studies (Torkashvand et al., 2024b; Torkashvand et al., 2024a; Torkashvand et al., 2023). Furthermore, they demonstrated the ability to control a wide range of *Salmonella* serotypes without affecting normal flora bacteria (Torkashvand et al., 2024a; Torkashvand et al., 2023). Here, we first evaluated the safety of the cocktail using the MTT assay in a human foreskin fibroblast cell line. Then, we examined the efficacy of phage cocktail in the elimination of *S. enteritidis* planktonic cells and biofilms via time-kill assay and crystal violet micro titer plate assay, respectively. In addition, we examined the antimicrobial activity of phage cocktail in various *Salmonella*-contaminated food products, and a 1-7-day-old chicken model infected with *S. enteritidis*. The latter was determined by measuring the beneficial effect of the cocktail on weight gain and mortality of the chickens.

## Methods and materials

### Bacteria strains and growth conditions

This study utilized two *S. enteritidis* strains, RVSRI 2293 with high virulence and ATCC13076, obtained from the Razi Vaccine and Serum Research Institute and the Department of Microbiology at the Faculty of Veterinary Medicine of Tehran University. The strains were stored at −80°C in 15% glycerol and cultured in Luria Bertani (LB) broth (LBB; Liofilchem®, Italy) or on LB agar under aerobic conditions at 37°C.

### Phages and their genome accession numbers

Our team initially isolated phages vB_SenS_TUMS_E4 (E4), vB_SenS_TUMS_E15 (E15), and vB_SenS_TUMS_E19 (E19) from various sources, including municipal, poultry, and hospital sewage. Recently, detailed information on the biological and genomic characteristics of these three bacteriophages has been made available (Torkashvand et al., 2024b; Torkashvand et al., 2024a; Torkashvand et al., 2023). Deposited in the National Center for Biotechnology Information Archive, the GenBank accession numbers are vB_SenS_TUMS_E4 (MZ955866.1), vB_SenS_TUMS_E15 (ON167532.1), and vB_SenS_TUMS_E19 (OL519843.1).

### Propagation and titration of phages

Phages were amplified in liquid media following a previously described method (Torkashvand et al., 2023), in which phages at a concentration of 10^10^ PFU/mL were added to a fluid culture of *S. enteritidis* ATCC 13076 with an optical density (OD) of 0.2 at 600 nm (~10^8^ CFU/mL). The mixtures were incubated overnight at 37°C with gentle shaking. After incubation, the samples were centrifuged at 10,000 × g for 10 min, and the resulting supernatants were filtered through 0.22 μm pore size filters. The filtered solution was stored at 4°C until further use. Phage titers were determined by diluting lysates 10-fold in phosphate-buffered saline (PBS) and plating the dilutions on 1% (w/v) Luria-Bertani (LB; Liofilchem ®, Italy) agar plates with a lawn of *S. enteritidis*.

### Preparation of the bacteriophage cocktail

To prepare the phage cocktail, each of the three phages was grown individually in LB broth, then centrifuged at 4°C at 10,000 × g for 10 min, and filtered through a low protein binding PES membrane filter (Membrane Solutions, United States) with a pore size of 0.22-μm. Lysates were kept at 4°C until further use. Each phage lysate underwent titration using the overlay technique. The lysate was diluted serially in phosphate-buffered saline (PBS), applied onto the corresponding host lawns, and incubated overnight at 37°C. The resulting plaques were counted and expressed as PFU/ml. The phage cocktail was created by combining the selected individual phages in equal proportions to achieve a final 10^10^ PFU/ml titer.

### The cell viability assay by MTT

The MTT colorimetric method was used to evaluate the cell viability of the phage-treated human foreskin fibroblasts (HFF) cell line. The tested cell line (10,000 cells/well) was incubated at 37°C for 24 h in 96-well plates and grown in Dulbecco’s modified Eagle’s medium/nutrient mixture F-12 (DMEM/F-12) supplemented with 1% (v/v) penicillin–streptomycin (5,000 U/mL), 20% (v/v) fetal bovine serum, and 2 mM L-glutamine under a 5% saturated CO2 atmosphere at 37°C. The phages and cocktail (100 μL) with a titer of 9.0 log PFU/mL were individually co-cultured with fibroblast cells (5 log cell/mL), followed by incubation in a 5% saturated CO2 atmosphere at 37°C for 24 h. Cell culture without a phage suspension was used as a control, and MTT was mixed with a final concentration of 0.5 mg/mL. Formazan crystals were formed by adding 100 μL of DMSO to each well, and the plates were incubated for 10 min. The light absorption of the solutions was then read at a wavelength of 570 nm using a spectrophotometer. The acquired optical densities of the treatment, control, and blank were entered into the following equation to determine the percentage cell viability (Rahimzadeh Torabi et al., 2021; Pelyuntha et al., 2022).

$$\mathrm{Viability}\;\left(\%\right)=\frac{\mathrm{Mean}\;{OD}_{\mathrm{treatment}}-\mathrm{Mean}\;{OD}_{\mathrm{blank}}}{\mathrm{Mean}\;{OD}_{\mathrm{control}}-\mathrm{Mean}\;{OD}_{\mathrm{blank}}}\times 100$$

### Time kill assay

*Salmonella enteritidis* was cultured to OD600 of 0.5 (~10^8^ CFU/mL) and diluted to a concentration of 10^7^ colony-forming units (CFU)/ml. Then, the phage cocktail solution was added to the host at a multiplicity of infection (MOI) of 0.01, 1, 100, 1,000 and 10,000, and samples were incubated at 37°C with moderate shaking. Bacterial growth in the fluid culture was monitored by quantifying the bacterial titer (cell count) at different points in time, and the CFU was calculated accordingly. LB medium was used as a negative control. The experiment was performed in triplicate (Kim et al., 2020).

### Phage treatment of *Salmonella* biofilms formed in 96-well microplate

A colorimetric method using a 96-well microplate was employed to quantitatively assess the effectiveness of bacteriophage treatment on the inhibition of biofilm formation and reduction of *Salmonella* preformed biofilms. In each well of the 96-well microplate, 100 μL of bacterial suspension was added at a final concentration of OD600 = 0.08–0.13 (1.5 × 10^8^ CFU/mL) was added. For the antibiofilm formation activity assay, bacteriophages and a phage cocktail (10^10^ PFU/mL) were added to the bacterial mixtures at an MOI of 100, followed by static incubation at 30°C for 24 h. For the reduction of preformed biofilm assay, *Salmonella* was initially incubated under the same conditions as described above for 24 h to allow biofilm formation, followed by bacteriophage and bacteriophage cocktail treatment for 4 h at MOI = 100. The minimum inhibitory concentration of ampicillin (2 μg/mL) and minimum biofilm eradication concentration of ampicillin (1,024 μg/mL) were loaded into the wells as positive controls. In the negative control, 100 μL of LB broth was added instead of 100 μL of the phage. Following bacteriophage treatment, each well was rinsed three times with sterile distilled water and allowed to air dry. Phage-treated biofilms in each well were stained with 1% crystal violet solution at 22°C for 45 min, eluted with 95% ethanol, and measured using a spectrometer at a wavelength of 600 nm. The percentage of biofilm reduction was calculated as follows: biofilm reduction% = (OD Control– OD Treatment)/OD Control × 100% (Gong and Jiang, 2017).

### Experimental design of the food productions

The efficacy of phage cocktail as a biocontrol agent in food has been evaluated in chicken breast, cherry tomatoes, quail eggshells, and lettuce, as these foods have been identified in multiple CDC reports as potential sources of *Salmonella* transmission (CDC, 2022; Whitworth, 2023; Johnston, 2023). The samples were purchased from a local supermarket then sliced aseptically in the laboratory. The samples were first checked for contamination with *Salmonella* spp., according to the WHO protocol (protocol number: 2010GFNLAB001). The samples were aseptically cut into 1 × 1 cm^2^ pieces and inoculated with *S. enteritidis* to a final viable count of 4 log10 CFU/cm^2^. After 30 min, phage cocktail was added at a final concentration of 10^8^ PFU/ cm^2^, and samples were incubated at 4°C or 25°C for 15–240 min. The samples were kept under sterile conditions in the center of the plate. For the recovery of bacterial loads from food products, 1 cm^2^ samples were collected at intervals of 0, 15, 30, 45, 60, 120 and 240 min during incubation. Each sample was then placed in a 2 mL Eppendorf tube with 1 mL of PBS buffer added under sterile conditions. The samples were homogenized using sterile bars and vortexed. To prevent the bacteriophage from being plated, 1 mL of the homogenized sample was centrifuged at 3000× g for 10 min to precipitate the bacteria, after which the supernatant containing the phage was discarded. Changes in bacterial viability counts for both control and experimental groups were assessed by adding 1 mL of PBS, followed by vigorous vortexing, serial dilution, and plating at each time point. CFU were determined using xylose lysine deoxycholate agar (XLD agar; Shahin et al., 2021). All experiments were performed in triplicates.

### Experimental design of the chick model

Ethical approval Animal care and study were performed according to the Guide for Care and Use of Laboratory Animals and the Animal Ethics Committees and Research Institutes of Tehran Medical University of Medical Sciences with identification code IR.TUMS.TIPS.REC.1399.013.

Experimental design: Eighty day-of-hatch chicks were obtained from a local hatchery and randomly divided into 4 groups of 20 birds (Table 1). Each group of birds was placed in cages, provided with feed and water *ad libitum*, and maintained at an age-appropriate temperature during the experiment.

**Table 1:** Scheme of experimental design.

Groups	No. of chicks	Time points of bacterial administrations (on day)	Doses of *S. enteritidis*	Treatment schedule of cocktail (days)	Doses of Cocktail	Time of euthanasia (day)
Negative control	20	–	–	–	–	14
Prophylactic	20	3	0.1 mL 10^8^ CFU/chick	2,3,4	0.5 mL 1.5 × 10^10^ PFU/chick	14
Treatment	20	3	0.1 mL 10^8^ CFU/chick	4,5,6	0.5 mL 1.5 × 10^10^ PFU/chick	14
Positive control	20	3	0.1 mL 10^8^ CFU/chick	–	–	14

In this study, since we aimed to evaluate the effectiveness of phage cocktail in reducing mortality in chicks infected with *Salmonella*, we used *S. enteritidis* RVSRI 2293, which has high virulence. The study included four groups of birds: negative control group (no challenges were applied in this group), treatment group (birds infected with *S. enteritidis* and receiving cocktail at 1.5 × 10^10^ PFU/ml), prophylactic group (birds receiving cocktail at 1.5 × 10^10^ PFU/ml and then infected with *S. enteritidis*), and positive control group (birds infected with *S. enteritidis*). To reach the final concentration loads (1.5 × 10^10^ PFU/ml) for the *in vivo* trial, phage suspensions were prepared via dilutions in PBS buffer, with the addition of 30% w/v of calcium carbonate (CaCO_3_), to prevent phage inactivation by the acidity of the chicken stomachs. 0.1 mL of the bacteriophage cocktail was administered through oral gavage on several occasions (Table 1). The targeted bacterium was the *Salmonella enterica* serovar Enteritidis RVSRI 2293 from the Razi Vaccine and Serum Research Institute collection. On day 3 of the trial, 0.1 mL of *S. enteritidis* was given to all birds directly through oral gavage at a dose of approximately 10^8^ CFU/mL, except for the negative control group. During this trial, the following parameters were recorded: pen body weight every alternate day, daily health records, illnesses, culls, and mortality, including the reasons for culls and probable causes of mortality.

### Statistical analysis

The analysis of experimental data was carried out using Graph Pad Prism 8.0.1. The one-way ANOVA model^1^ assessed significant differences among various conditions and tested parameters. The results are expressed as the mean ± standard error, and the statistical significance level was set at *p* ≤ 0.0001.

## Results

### The cell viability assay by MTT

**Figure 1:**
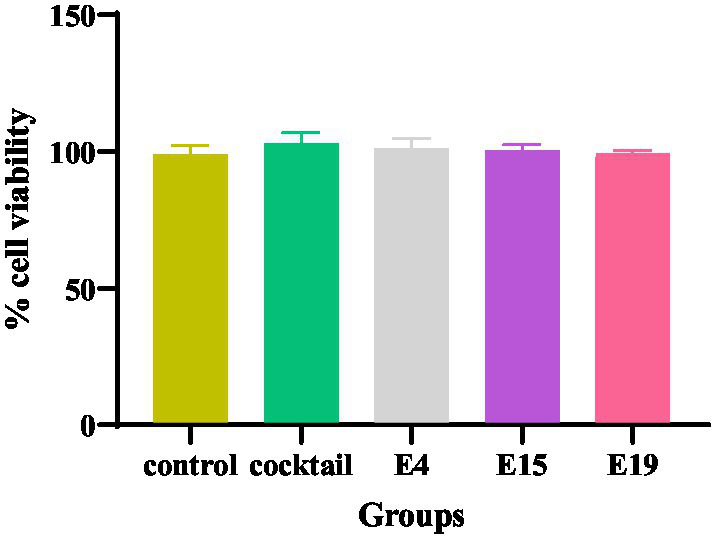
The cytotoxic effect of phages on the HFF cell line.

The findings regarding the human foreskin fibroblast (HFF) cells indicate a significantly high cell viability across all experimental groups, including the control group and those exposed to specific phages (E4, E15, E19, and phage cocktail). Notably, the highest percentage of cell viability was recorded at the 24-h mark for all groups tested (*p* < 0.0001; Figure 1).

### Time kill assay

**Figure 2:**
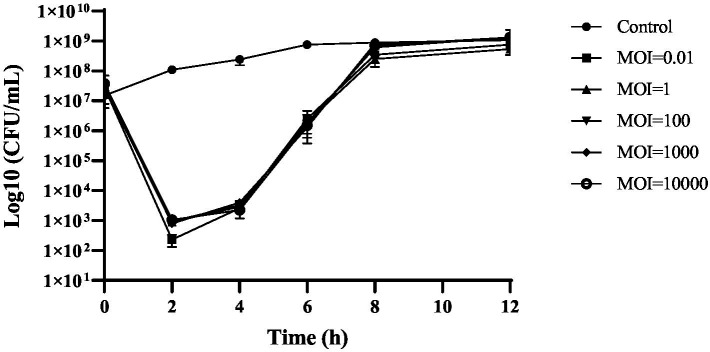
Kinetics of lytic activity of phage cocktail against *S. enteritidis* ATCC 13076 at different MOIs.

The susceptibility of planktonic cells to phage cocktail was measured using a time-kill kinetics test. Within 2 h, employing MOIs of 0.01, 1, 100, 1,000, and 10,000 led to a 4 Log decline in bacterial levels to below detectable limits (<1 CFU/10 μL; *p* < 0.0001). Bacterial levels were identical across different MOIs, suggesting that the concentration of the phage cocktail did not have any impact on its ability to kill bacteria (Figure 2). In the initial 10 h of testing, colony counts fell sharply at all MOIs compared with the control (*p* < 0.0001). However, they started to increase 4 h later, potentially owing to the emergence of resistance.

### Lytic activity of isolated phages and phage cocktail in preventing biofilm formation caused by *Salmonella enteritidis*

**Figure 3:**
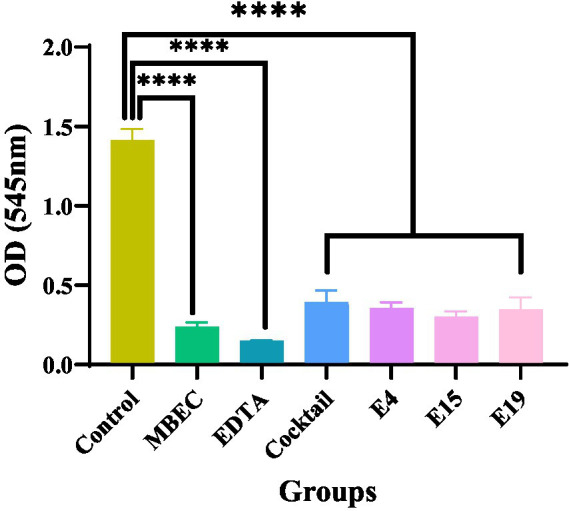
The effect of phages on the prevention of biofilm formation.

This test investigated the lytic activity of E4, E15, and E19 phages individually, as well as in a phage cocktail (pfu/ml 10^10^), compared to the antibiotic control with the minimum biofilm eradication concentration (MBEC) (μg/ml 1,024) in inhibiting biofilm formation within 24 h. The results showed that both the phage and phage cocktail, similar to the antibiotic control, significantly reduced biofilm formation after 24 h compared with the control (*p* ≤ 0.0001; Figure 3).

### Lytic activity of isolated phages and phage cocktail in destroying biofilm caused by *Salmonella enteritidis*

**Figure 4:**
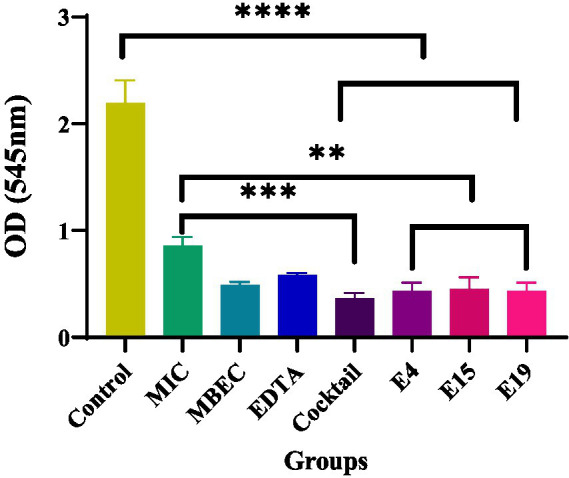
The effect of phages on the eradication of biofilm.

The lytic activity of E4, E15, and E19 phages was evaluated at 24 h both separately and as a phage cocktail (10^10^ pfu/ml) and compared to the antibiotic control with the minimum biofilm eradication concentration (MBEC) (μg/ml1024) and the minimum inhibitory concentration (MIC) (μg/ml). Separate phages and phage cocktail showed better efficacy in biofilm destruction after 24 h compared to the antibiotic control with minimum inhibitory concentrations (*p* ≤ 0.0016 and *p* ≤ 0.0003; Figure 4). The effectiveness of phage cocktail in destroying biofilms was higher than that of phages individually and antibiotics at different concentrations.

### Antibacterial efficacy of phage cocktail on food samples

**Figure 5:**
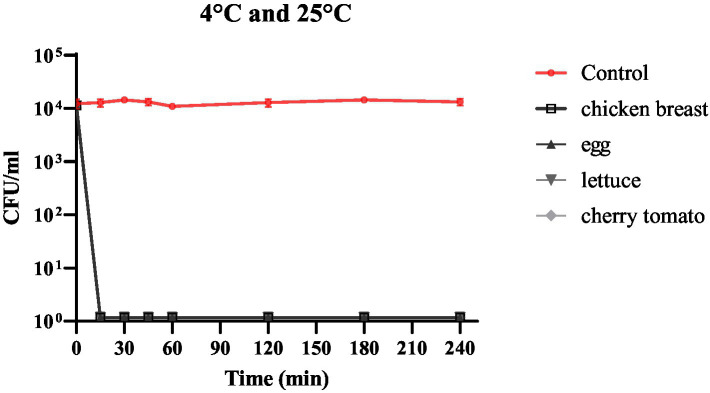
The impact of phage cocktail treatment on the number of viable *S. enteritidis* cells in chicken breast, quail eggshell, cherry tomato, and lettuce samples over a 240-min period at both 4°C and 25°C.

The efficacy of phage cocktail was measured in terms of its ability to inhibit bacterial growth in raw chicken breast, quail eggshell, cherry tomato, and lettuce infected with *S. enteritidis*. The bacterial solution and phage cocktail were added to the samples at an MOI of 10,000. The MOI was selected for this experiment based on previous reports (Islam et al., 2020; Ssekatawa et al., 2021). In addition, one of the significant advantages of utilizing this phage cocktail is the ability to apply a high multiplicity of infection (MOI) without requiring additional concentration of the phages. This characteristic is particularly beneficial as it allows for more efficient targeting of bacterial pathogens, enhancing the likelihood of successful bacterial lysis. In food samples, phage cocktail fully reduced viable *S. enteritidis* at both 4°C and 25°C within 15 min, in spite of the food model (*p* < 0.0001; Figure 5).

### The effect of phage cocktail on *Salmonella* infection in 1-day-old chicks

**Figure 6:**
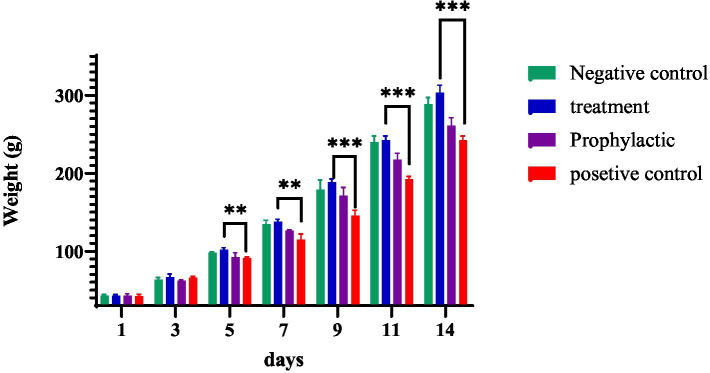
The effect of phage cocktail on the growth of birds up to the age of 14 days.

During the 14-day maintenance period, the weights of the chicks were measured every other day. There was a significant increase in weight gain in the treatment group compared to that of the untreated group (positive control), which remained significant until the end of the period (*p* ≤ 0.0002; Figure 6). Animals were examined daily for clinical symptoms and mortality. No casualties were recorded in the treatment group; however, in the positive control group, 10% mortality was observed.

## Discussion

Bacteriophages have attracted great interest as biocontrol agents that can replace antibiotics, chemical preservatives, and sanitizers in the food industry. Nonetheless, their integration into food-processing protocols requires additional studies to explore their safety and efficacy. In line with the farm-to-fork approach, we carried out a comprehensive set of tests designed to determine the safety and effectiveness of a new phage cocktail, which yielded encouraging results that are discussed in detail below.

The results from our time-kill assay demonstrated that in the initial hours, the number of *Salmonella* planktonic cells declined substantially, independent of the employed MOI. This result is contrary to that of a previously published review, implying that the effectiveness of phage-induced bacterial lysis is a direct function of the MOI (Jorda et al., 2023). This may be rooted in the replicative nature of phages, leading to their rapid propagation in liquid media. However, some hours later, we observed that the bacterial count started to increase, most likely stemming from the emergence of resistance. However, based on earlier studies, phage-resistant bacteria are not as troublesome as expected because they are more readily eliminated by phagocytes and the innate immune system; therefore, the emergence of phage resistance should not be regarded as a formidable obstacle (Gu et al., 2012; Kamyab et al., 2023).

In our previous studies, the effects of phages E4, E15, and E19 were examined separately for normal flora bacteria and other bacteria, and the results indicated high specificity of the phages. Phages E4, E15, and E19 had no effect on normal flora bacteria, whereas they lysed all laboratory strains of *Salmonella* with a high EOP (Torkashvand et al., 2023; Torkashvand et al., 2024a). The lack of lytic activity against normal flora bacteria indicates that these phages can selectively target pathogenic strains without disrupting beneficial microbial communities essential for human health. This specificity is crucial as it minimizes the risk of dysbiosis, a condition in which the balance of microbial populations is disturbed, potentially leading to adverse health outcomes. These findings align with the growing interest in phage therapy as a more precise alternative to broad-spectrum antibiotics, which can have far-reaching consequences for the microbiome (Bozidis et al., 2024). Furthermore, the high efficiency of plating (EOP) observed with laboratory strains of *Salmonella* points to the potential effectiveness of these phages in real-world applications. Future studies should focus on evaluating the efficacy of this phage cocktail against diverse *Salmonella* serotypes in various food matrices under different environmental conditions. Additionally, exploring the mechanisms behind the phage-host interactions may yield insights into optimizing phage formulations for enhanced lytic activity.

The safety of phage cocktails is a critical consideration, especially when investigating their effects on food products that may be consumed raw. To assess this safety, the MTT test is utilized to evaluate the impact of phages on human cell lines. Additionally, in the context of phage therapy for broiler chickens, it is essential to ensure that the phage cocktail is safe for individuals who work directly with the animals. This dual focus on safety for both food consumers and agricultural workers underscores the importance of thorough testing in phage therapy applications. Our findings derived from the *in vitro* cell viability assay revealed that phage cocktail is non-toxic to human fibroblast cells, and thus, foods treated beforehand with this cocktail can be considered innocuous for its consumers. Surprisingly, most studies regarding *Salmonella* phages as biocontrol agents have concentrated on the evaluation of efficacy, whereas safety assessments have often been overlooked (Almutairi et al., 2022). The small number of studies in this area is discussed as follows: Two studies have probed the safety of Bafasal®, a food additive comprising four *Salmonella* phages. They ran a series of experiments and concluded that Bafasal® carries no risk to consumers, avian species, or the environment (Bampidis et al., 2021), (Wojcik et al., 2020). Furthermore, Li et al. (2020) orally administered a determined dose of a single phage to a group of mice for 14 days; no adverse effects were observed in the examined animals. Similarly, Korzeniowski et al. (2022) tested the toxicity of two *Salmonella* phages on chicken fibroblasts for up to 120 h and no decrease in cell viability was observed at any tested concentration. Likewise, Pelyuntha et al. (2022) exposed human fibroblasts and Caco-2 cells to three *Salmonella* phages for 72 h and reported a high cell viability of nearly 100% regardless of phage concentration.

*Salmonella* biofilms are a serious threat to food safety as they facilitate bacterial colonization, persistence, and survival on industrial equipment, contributing to later food contamination (Mkangara, 2023). To address this issue, various studies have been conducted to investigate the effectiveness of phages in biofilm removal. Korzeniowski et al. examined the ability of phages alone and as cocktails to destroy biofilms formed by *S. enteritidis* on a 96-well microplate and a stainless-steel surface which resulted in a significant reduction of biofilm in both cases. Likewise, Duc et al. employed a single phage against biofilms formed by *S. enteritidis* and *S. typhimurium* on polystyrene, stainless steel, and cabbage leaves, and obtained fairly good efficacy in biofilm removal. Additionally, Ge et al. applied a single phage against biofilms formed by *S. enteritidis* and *S.* Pullorum on a metal surface and attained the destruction of the biofilm in 1 h. While evaluation of phage efficacy in biofilm disruption alone is helpful, it is also worthwhile to compare the effectiveness of phages to that of antibiotics because the ultimate goal is to substitute antibiotics with phages in the food production process. In the present study, phage cocktail exhibited similar efficacy to the antibiotic control in the prevention of biofilm formation and notably, significantly better efficacy compared to the antibiotic control at both MIC and MBEC in the destruction of the previously formed biofilm. Our results were in accordance with those of a study conducted by Kosznik-Kwasnicka et al. (2022), indicating that *Salmonella* phages were either as effective as antibiotics or even more effective than them in terms of their anti-biofilm activity. Also, the results obtained from the 24-h biofilm can serve as a basis for future research where longer times, such as 72 h, will be examined.

Several studies have reported a favorable reduction in *Salmonella* counts in experimental food models following phage treatment (Khan and Rahman, 2022a; Khan and Rahman, 2022b). Here, we briefly discuss the most recent studies on this topic. In a study carried out by Duc et al. (2023) a single phage was applied over cabbage leaves pre-contaminated with *S. enteritidis* and *S. typhimurium*, which resulted in a significant reduction of viable bacteria at both 24°C and 4°C. Similarly, Islam et al. (2019) first contaminated chicken breast and pasteurized milk with *S. enteritidis* and *S. typhimurium* and then treated them with a cocktail of three phages. In both food samples, the cocktail fully eliminated *Salmonella* in less than 6 h. Gvaladze et al. (2024) artificially contaminated chicken skin and sprayed a six-phage cocktail onto it, resulting in a 1.8 log decrease in *S. enteritidis* in 30 min. Rivera et al. (2022) applied a single phage to contaminated chicken breast and achieved a 2.5 log reduction in *S. enteritidis* at 4°C. Sritha and Bhat (2021) treated four food samples, namely, liquid egg, eggshell, milk, and lettuce, with a four-phage cocktail, which led to a considerable decrease in *Salmonella* spp. at 4°C and 28°C. Our study differed from previous studies in that complete eradication of *S. enteritidis* was observed in all four food matrices in just 15 min at both 25°C and 4°C. The variations in results might be due to factors such as extent of lytic activity and specificity of the applied phages, differences in phage concentration, delivery method, and growth conditions affecting the physiological state of the host, just to mention a few (Almutairi et al., 2022; Bao et al., 2015).

Findings from previous studies using *Salmonella*-infected animal models have revealed that phage therapy can successfully reduce bacterial colonization in the gastrointestinal tract of chickens and, therefore, lower the prevalence of *Salmonella* among poultry flocks (Pelyuntha et al., 2022; Thanki et al., 2023). Moreover, salmonellosis can increase mortality in poultry farms, especially in young birds (Shaji et al., 2023). In light of these reports, we conducted an *in vivo* experiment to determine the effect of phage cocktail treatment on *Salmonella* colonization and mortality rate in experimentally infected chicks. However, given the extremely high number of *E. coli* colonies in the collected fecal samples and the lack of a strain of *S. enteritidis* with antibiotic resistance selective markers at our disposal, we were not able to accurately determine *Salmonella* counts in fecal cultures. In addition, the results from the mortality rate assessments were not quite pronounced, although 10% mortality was observed in the positive control group, and none was observed in the treatment groups. Interestingly, however, we noticed that feeding broilers with phage cocktail significantly improved their weight gain compared to the control group. In agreement with our results, Sarrami et al. (2022) compared the effects of the addition of ProBe-Bac, a phage cocktail against *Salmonella* and *E. coli*, with the addition of colistin, an antibiotic, to the diet of a group of bacterially unchallenged broilers. Their results showed higher body weight gain and lower feed conversion ratio (FCR) in phage-fed chickens than in those who received colistin treatment. Similarly, Upadhaya et al. (2021) gave two different concentrations of a phage cocktail against *Salmonella* and *E. coli* as dietary supplements to two poultry flocks under normal physiological conditions and reported increased weight gain in both phage-treated groups compared with the control. The weight-promoting properties of bacteriophages can be explained by the fact that anorexia and severe watery diarrhea are common symptoms of salmonellosis in birds, which in turn lead to less food intake and loss of nutrients, resulting in less overall weight gain (Tariq et al., 2022). Hence, the addition of effective phage cocktails to the diet of broiler chickens can prevent salmonellosis and consequently boost weight gain. One limitation of this experiment is that owing to the lack of adequate space for older chickens in our animal husbandry, we needed to restrict the duration of our study to 14 days. Therefore, future research is needed to undertake the above assessments on broiler chickens until they reach the average slaughter age, that is 30–35 days.

## Conclusion and future work

In summary, our comprehensive evaluation of phage cocktail as a biocontrol agent against *Salmonella* yielded promising results regarding both its efficacy and safety. Importantly, the specificity of phages E4, E15, and E19 for *Salmonella*, without affecting normal flora, underscores their potential as targeted alternatives to traditional antibiotics, thereby preserving the integrity of beneficial microbial communities. Moreover, the non-toxicity of phage cocktail to human fibroblast cells reinforces its suitability for application in food processing. This finding is particularly relevant, given the growing consumer demand for safe and effective food preservation methods. While existing literature has often prioritized efficacy over safety in the evaluation of phage applications, our study contributes to a more balanced understanding by demonstrating that phage cocktail poses no risk to human health. Further studies are warranted to assess the performance of this phage cocktail against a broader range of *Salmonella* serotypes in various food environments. Additionally, understanding the mechanisms of phage-host interactions is essential for optimizing phage formulations. Ultimately, our findings support the integration of bacteriophages into food processing protocols as a viable strategy for enhancing food safety while minimizing reliance on antibiotics and chemical preservatives.

## Data Availability

The datasets presented in this study can be found in online repositories. The names of the repository/repositories and accession number(s) can be found at: https://www.ncbi.nlm.nih.gov/genbank/, NC_073179.1; https://www.ncbi.nlm.nih.gov/genbank/, ON167532.1; https://www.ncbi.nlm.nih.gov/genbank/, NC_073178.1.

## Footnotes

^1^https://www.graphpad.com/

## References

[ref1] Almutairi M. Imam M. Alammari N. Hafiz R. Patel F. Alajel S. (2022). Using phages to reduce Salmonella prevalence in chicken meat: a systematic review. Phage (New Rochelle) 3, 15–27. doi: 10.1089/phage.2021.0017, PMID: 36161190 PMC9041517

[ref2] Bampidis V. Azimonti G. Bastos M. L. Christensen H. Dusemund B. Kouba M.. (2021). Safety and efficacy of a feed additive consisting on the bacteriophages PCM F/00069, PCM F/00070, PCM F/00071 and PCM F/00097 infecting *Salmonella gallinarum* B/00111 (Bafasal®) for all avian species (Proteon pharmaceuticals S.A.). EFSA J. 19:e06534. doi: 10.2903/j.efsa.2021.6534, PMID: 34025802 PMC8127046

[ref3] Bao H. Zhang P. Zhang H. Zhou Y. Zhang L. Wang R. (2015). Bio-control of *Salmonella* Enteritidis in foods using bacteriophages. Viruses 7, 4836–4853. doi: 10.3390/v7082847, PMID: 26305252 PMC4576208

[ref4] Bozidis P. Markou E. Gouni A. Gartzonika K. (2024). Does phage therapy need a Pan-phage? Pathogens 13:522. doi: 10.3390/pathogens13060522, PMID: 38921819 PMC11206709

[ref5] CDC. (2022). *Salmonella* and Food. Available at: https://www.cdc.gov/foodsafety/communication/salmonella-food.html.

[ref6] Clavijo V. Morales T. Vives-Flores M. J. Reyes Muñoz A. (2022). The gut microbiota of chickens in a commercial farm treated with a *Salmonella* phage cocktail. Sci. Rep. 12:991. doi: 10.1038/s41598-021-04679-6, PMID: 35046416 PMC8770602

[ref7] Duc H. M. Zhang Y. Son H. M. Huang H. H. Masuda Y. Honjoh K. I.. (2023). Genomic characterization and application of a novel bacteriophage STG2 capable of reducing planktonic and biofilm cells of *Salmonella*. Int. J. Food Microbiol. 385:109999. doi: 10.1016/j.ijfoodmicro.2022.109999, PMID: 36335891

[ref8] Gong C. Jiang X. (2017). Application of bacteriophages to reduce *Salmonella* attachment and biofilms on hard surfaces. Poult. Sci. 96, 1838–1848. doi: 10.3382/ps/pew463, PMID: 28339743

[ref9] Gu J. Liu X. Li Y. Han W. Lei L. Yang Y.. (2012). A method for generation phage cocktail with great therapeutic potential. PLoS One 7:e31698. doi: 10.1371/journal.pone.0031698, PMID: 22396736 PMC3291564

[ref10] Gvaladze T. Lehnherr H. Hertwig S. (2024). A bacteriophage cocktail can efficiently reduce five important *Salmonella* serotypes both on chicken skin and stainless steel. Front. Microbiol. 15:1354696. doi: 10.3389/fmicb.2024.1354696 38500580 PMC10944927

[ref11] Islam M. S. Zhou Y. Liang L. Nime I. Liu K. Yan T.. (2019). Application of a phage cocktail for control of *Salmonella* in foods and reducing biofilms. Viruses 11:841. doi: 10.3390/v11090841, PMID: 31510005 PMC6784009

[ref12] Islam M. S. Zhou Y. Liang L. Nime I. Yan T. Willias S. P.. (2020). Application of a broad range lytic phage LPST94 for biological control of *Salmonella* in foods. Microorganisms 8:247. doi: 10.3390/microorganisms8020247, PMID: 32069865 PMC7074677

[ref13] Johnston L. (2023). *Salmonella* and tomatoes. The produce contamination problem. Amsterdam, Netherlands: Elsevier.

[ref14] Jorda J. Lorenzo-Rebenaque L. Montoro-Dasi L. Marco-Fuertes A. Vega S. Marin C. (2023). Phage-based biosanitation strategies for minimizing persistent Salmonella and Campylobacter Bacteria in poultry. Animals (Basel) 13:826. doi: 10.3390/ani13243826, PMID: 38136863 PMC10740442

[ref15] Kamyab H. Torkashvand N. Shahverdi A. R. Khoshayand M. R. Sharifzadeh M. Sepehrizadeh Z. (2023). Isolation, characterization, and genomic analysis of vB_PaeS_TUMS_P81, a lytic bacteriophage against *Pseudomonas aeruginosa*. Virus Genes 59, 132–141. doi: 10.1007/s11262-022-01954-0, PMID: 36357763

[ref16] Khan M. Rahman S. R. (2022). Use of phages to treat antimicrobial-resistant Salmonella infections in poultry. Vet. Sci. 9:438. doi: 10.3390/vetsci9080438, PMID: 36006353 PMC9416511

[ref17] Kim S. Lee D.-W. Jin J.-S. Kim J. (2020). Antimicrobial activity of LysSS, a novel phage endolysin, against *Acinetobacter baumannii* and *Pseudomonas aeruginosa*. J. Glob. Antimicrob. Resist. 22, 32–39. doi: 10.1016/j.jgar.2020.01.005 32006750

[ref18] Korzeniowski P. Sliwka P. Kuczkowski M. Misic D. Milcarz A. Kuzminska-Bajor M. (2022). Bacteriophage cocktail can effectively control *Salmonella* biofilm in poultry housing. Front. Microbiol. 13:901770. doi: 10.3389/fmicb.2022.901770 35847069 PMC9277115

[ref19] Kosznik-Kwasnicka K. Stasilojc M. Grabowski L. Zdrojewska K. Wegrzyn G. Wegrzyn A. (2022). Efficacy and safety of phage therapy against *Salmonella enterica* serovars Typhimurium and Enteritidis estimated by using a battery of in vitro tests and the galleria mellonella animal model. Microbiol. Res. 261:127052. doi: 10.1016/j.micres.2022.127052 35533436

[ref20] Lavilla M. Domingo-Calap P. Sevilla-Navarro S. Lasagabaster A. (2023). Natural killers: opportunities and challenges for the use of bacteriophages in microbial food safety from the one health perspective. Food Secur. 12:552. doi: 10.3390/foods12030552, PMID: 36766081 PMC9914193

[ref21] Li M. Lin H. Jing Y. Wang J. (2020). Broad-host-range *Salmonella* bacteriophage STP4-a and its potential application evaluation in poultry industry. Poult. Sci. 99, 3643–3654. doi: 10.1016/j.psj.2020.03.051, PMID: 32616261 PMC7597861

[ref22] Mkangara M. (2023). Prevention and control of human *Salmonella enterica* infections: an implication in food safety. Int. J. Food Sci. 2023:8899596. doi: 10.1155/2023/8899596, PMID: 37727836 PMC10506869

[ref23] Pelyuntha W. Yafa A. Ngasaman R. Yingkajorn M. Chukiatsiri K. Champoochana N.. (2022). Oral administration of a phage cocktail to reduce *Salmonella* colonization in broiler gastrointestinal tract-a pilot study. Animals (Basel) 12:87. doi: 10.3390/ani12223087, PMID: 36428315 PMC9686501

[ref24] Rahimzadeh Torabi L. Doudi M. Naghavi N. S. Monajemi R. (2021). Bacteriophages PɸEn-CL and PɸEn-HO can eliminate MDR *Enterobacter* cloacae and *Enterobacter hormaechei* isolated from burn wound infections without toxicity for human skin cells. FEMS Microbiol. Lett. 368:fnab143. doi: 10.1093/femsle/fnab143, PMID: 34849758

[ref25] Rivera D. Moreno-Switt A. I. Denes T. G. Hudson L. K. Peters T. L. Samir R.. (2022). Novel *Salmonella* phage, vB_Sen_STGO-35-1, characterization and evaluation in chicken meat. Microorganisms 10:606. doi: 10.3390/microorganisms10030606, PMID: 35336181 PMC8954984

[ref26] Sarrami Z. Sedghi M. Mohammadi I. Kim W. K. Mahdavi A. H. (2022). Effects of bacteriophage supplement on the growth performance, microbial population, and PGC-1alpha and TLR4 gene expressions of broiler chickens. Sci. Rep. 12:14391. doi: 10.1038/s41598-022-18663-1, PMID: 35999253 PMC9399175

[ref27] Shahin K. Zhang L. Delfan A. S. Komijani M. Hedayatkhah A. Bao H.. (2021). Effective control of *Shigella* contamination in different foods using a novel six-phage cocktail. LWT 144:111137. doi: 10.1016/j.lwt.2021.111137

[ref28] Shaji S. Selvaraj R. K. Shanmugasundaram R. (2023). *Salmonella* infection in poultry: a review on the pathogen and control strategies. Microorganisms 11:2814. doi: 10.3390/microorganisms11112814, PMID: 38004824 PMC10672927

[ref29] Sritha K. S. Bhat S. G. (2021). In vitro efficiency evaluation of phage cocktail for biocontrol of *Salmonella* spp. in food products. Arch. Microbiol. 203, 5445–5452. doi: 10.1007/s00203-021-02522-0, PMID: 34406443

[ref30] Ssekatawa K. Byarugaba D. K. Kato C. D. Wampande E. M. Ejobi F. Tweyongyere R.. (2021). A review of phage mediated antibacterial applications. Alex. J. Med. 57, 1–20. doi: 10.1080/20905068.2020.1851441

[ref31] Tariq S. Samad A. Hamza M. Ahmer A. Muazzam A. Ahmad S.. (2022). *Salmonella* in poultry; an overview Int. J. Multidiscip. Sci. Arts 1, 80–84. doi: 10.47709/ijmdsa.v1i1.1706

[ref32] Teklemariam A. D. Al-Hindi R. R. Albiheyri R. S. Alharbi M. G. Alghamdi M. A. Filimban A. A.. (2023). Human salmonellosis: a continuous global threat in the farm-to-fork food safety continuum. Food Secur. 12:1756. doi: 10.3390/foods12091756, PMID: 37174295 PMC10178548

[ref33] Thanki A. M. Hooton S. Gigante A. M. Atterbury R. J. Clokie M. R. (2021). Potential roles for bacteriophages in reducing Salmonella from poultry and swine. *Salmonella* spp.-a global challenge. London: IntechOpen.

[ref34] Thanki A. M. Hooton S. Whenham N. Salter M. G. Bedford M. R. O'Neill H. V. M.. (2023). A bacteriophage cocktail delivered in feed significantly reduced Salmonella colonization in challenged broiler chickens. Emerg. Microbes Infect. 12:2217947. doi: 10.1080/22221751.2023.2217947, PMID: 37224439 PMC10283443

[ref35] Torkashvand N. Kamyab H. Shahverdi A. R. Khoshayand M. R. Karimi Tarshizi M. A. Sepehrizadeh Z. (2024a). Characterization and genome analysis of a broad host range lytic phage vB_SenS_TUMS_E19 against *Salmonella enterica* and its efficiency evaluation in the liquid egg. Can. J. Microbiol. 70, 358–369. doi: 10.1139/cjm-2024-0013, PMID: 38990097

[ref36] Torkashvand N. Kamyab H. Shahverdi A. R. Khoshayand M. R. Karimi Tarshizi M. A. Sepehrizadeh Z. (2024b). Whole-genome sequencing of *Salmonella* phage vB_SenS_TUMS_E15 for bio-control in the food chain. Acta Biochim. Iran. 2, 31–35. doi: 10.18502/abi.v2i1.16245

[ref37] Torkashvand N. Kamyab H. Shahverdi A. R. Khoshayand M. R. Sepehrizadeh Z. (2023). Isolation, characterization, and genome analysis of a broad host range *Salmonella* phage vB_SenS_TUMS_E4: a candidate bacteriophage for biocontrol. Vet. Res. Commun. 47, 1493–1503. doi: 10.1007/s11259-023-10105-1 37097546

[ref38] Upadhaya S. D. Ahn J. M. Cho J. H. Kim J. Y. Kang D. K. Kim S. W.. (2021). Bacteriophage cocktail supplementation improves growth performance, gut microbiome and production traits in broiler chickens. J. Anim. Sci. Biotechnol. 12:49. doi: 10.1186/s40104-021-00570-6, PMID: 33858501 PMC8050931

[ref39] Whitworth J. (2023). *Nearly 150 sick in Salmonella outbreak affecting 11 countries*. Available at: https://www.foodsafetynews.com/2023/11/nearly-150-sick-in-salmonella-outbreak-affecting-11-countries/.

[ref40] Wojcik E. A. Stanczyk M. Wojtasik A. Kowalska J. D. Nowakowska M. Lukasiak M.. (2020). Comprehensive evaluation of the safety and efficacy of BAFASAL((R)) bacteriophage preparation for the reduction of *Salmonella* in the food chain. Viruses 12:742. doi: 10.3390/v12070742, PMID: 32664206 PMC7412135

[ref41] World Health Organization. (2016). Interventions for the control of non-typhoidal *Salmonella* spp. in beef and pork: meeting report and systematic review. Geneva: World Health Organization.

[ref42] Xu Y. (2021). Phage and phage lysins: new era of bio-preservatives and food safety agents. J. Food Sci. 86, 3349–3373. doi: 10.1111/1750-3841.15843, PMID: 34302296

